# Retinal Organisation and Systemic Vascular Changes Assessed by Adaptive Optics and Doppler Ultrasonography Following Anti-VEGF Therapy in Patients with Diabetic Macular Oedema

**DOI:** 10.3390/biomedicines14010124

**Published:** 2026-01-08

**Authors:** Janusz Pieczyński, Arleta Berlińska, Joanna M. Harazny

**Affiliations:** 1 Department of Ophthalmology, Faculty of Medicine, University of Warmia and Mazury in Olsztyn, 10-719 Olsztyn, Poland; 2 Ophthalmology Department, Provincial Specialist Hospital in Olsztyn, 10-561 Olsztyn, Poland; aberlinska@wss.olsztyn.pl; 3 Department of Physiology and Pathophysiology, Faculty of Medicine, University of Warmia and Mazury in Olsztyn, 10-719 Olsztyn, Poland; joanna.harazna@uwm.edu.pl; 4 Department of Nephrology and Hypertension, University Hospital of the University of Erlangen-Nuremberg, 91054 Erlangen, Germany

**Keywords:** diabetic macular oedema, anti-VEGF, adaptive optics, carotid ultrasound, central hemodynamics, augmentation index, pulse wave velocity, retinal microcirculation, wall-to-lumen ratio, cone regularity

## Abstract

**Objective:** Evaluate the efficacy and safety following intravitreal anti-vascular endothelial growth factor (anti-VEGF) therapy in patients with diabetic macular oedema (DME). **Methods:** To evaluate retinal microvascular remodelling and photoreceptor metrics using adaptive optics (AO) alongside systemic vascular status assessed by brachial/aortic hemodynamic and carotid ultrasound. We conducted a single-centre longitudinal study including twenty-one patients with DME. The following four diagnostic visits were performed: baseline (V1, no anti-VEGF treatment), 2–3 months (V2), 6–8 months (V3), and 12–14 months (V4). Adaptive optics (rtx1) measured foveal cone number (N) and regularity (Reg) within a standardised 80 × 80 µm window, and superior temporal retinal arteriole morphology after the first bifurcation (vessel diameter [VD], lumen diameter [LD], wall thickness [WT], wall-to-lumen ratio [WLR], and wall cross-sectional area [WCSA]). SphygmoCor provided peripheral (brachial) and central (aortic) pressures, augmentation pressure (AP), augmentation index (AIx), and carotid–femoral pulse wave velocity (PWV and PWVHR heart rate adjusted). Carotid ultrasound assessed intima–media thickness (IMT), carotid lumen diameter (CLD), and IMT/CLD ratio (IMTLR) 2 mm proximal to the bifurcation in diastole. Visual acuity (Visus), intraocular pressure (IOP), and central retinal thickness (CRT) were obtained at each visit. **Results:** In the treated eye (TE), WLR showed a significant overall change (Friedman *p* = 0.007), with a modest V4 vs. V1 increase (Wilcoxon *p* = 0.045); LD also varied across visits (Friedman *p* = 0.034). Cone metrics improved as follows: Reg increased over time (Friedman *p* = 0.019), with a significant rise at V4 vs. V1 (*p* = 0.018), and cone number increased at V3 vs. V1 (*p* = 0.012). Functional/structural outcomes improved as follows: visual acuity increased at V3 (*p* = 0.009) and V4 (*p* = 0.028), while CRT decreased at V3 (*p* = 0.002) and V4 (*p* = 0.030); IOP remained stable compared to V1. Systemic hemodynamics was largely unchanged; small fluctuations in DBP and cDBP across V1–V4 were observed (Friedman *p* = 0.034 and *p* = 0.022, respectively), whereas AIx, AP, PWV, and PWVHR showed no significant trends. Carotid IMT, CLD, and IMTLR did not change significantly across visits, supporting systemic vascular safety. **Conclusions:** Intravitreal anti-VEGF therapy in DME was associated with improvements in photoreceptor organisation and macular structure/function, with AO-derived arteriolar remodelling detectable over time, and no adverse changes in large-artery structure. These findings support ocular efficacy and systemic vascular safety; confirmation in larger cohorts is warranted.

## 1. Introduction

Diabetic macular oedema (DME) is a leading cause of vision loss in working-age adults and arises from the breakdown of the inner blood–retina barrier and microvascular dysfunction.

Intravitreal inhibitors of vascular endothelial growth factor (anti-VEGF) are standard of care for centre-involving DME and improve visual acuity and retinal thickness in randomised trials [1,2,3,4]. However, concerns persist regarding potential systemic effects after repeated intravitreal dosing, particularly in patients with cardiovascular risk factors, given that low systemic exposure might still modulate arterial stiffness or wave reflection indices [5,6,7].

At the same time, adaptive optics (AO) enables in vivo visualisation of human photoreceptors and precise quantification of retinal microvessels, offering sensitive biomarkers of microvascular remodelling beyond conventional fundus imaging or OCT [8,9,10,11].

Combining AO with non-invasive assessments of central hemodynamics (SphygmoCor-derived central blood pressure, augmentation pressure, and index) and carotid ultrasound (intima–media thickness and lumen diameter) provides a comprehensive multiscale view—from photoreceptors and arterioles to large elastic arteries—within a single longitudinal protocol [12,13,14,15,16].

We hypothesised that intravitreal anti-VEGF drugs (aflibercept or bevacizumab) would improve retinal structure followed by function (e.g., visual acuity) improvement and photoreceptor organisation, while systemic vascular parameters, including carotid morphology and aortic stiffness, would remain stable. To test this, we conducted a single-centre observational study with four diagnostics, planned visits over 12–14 months, integrating AO imaging, optical coherence tomography (OCT), central hemodynamics, and carotid ultrasound in patients with DME undergoing regular anti-VEGF therapy.

## 2. Materials and Methods

### 2.1. Study Design and Population

This single-centre, observational study was conducted at the Department of Ophthalmology, Provincial Specialist Hospital, Olsztyn, Poland. The protocol was approved by the Bioethics Committee of the University of Warmia and Mazury in Olsztyn (approval no. 44/2017). All participants received verbal and written information about the study and provided written informed consent; personal data were anonymized. We enrolled 22 patients with DME eligible for intravitreal anti-VEGF; 1 patient discontinued after enrolment. The final analysis included 21 individuals aged 29–84 years (mean 64.95 ± 12.71; median 68; and IQR 59–72.5), 11 women (52.4%) and 10 men (47.6%). Diabetes types were type 1 (*n* = 4), type 2 (*n* = 17), and a history of gestational diabetes (*n* = 1). All patients had diabetic retinopathy; twenty had prior laser treatment.

### 2.2. Inclusion Criteria

- Presence of cystoid macular oedema confirmed by optical coherence tomography (OCT);
- Eligibility for anti-VEGF therapy regardless of prior laser treatment history;
- No previous anti-VEGF treatment;
- Transparent optical media;
- Age over 18 years old.

### 2.3. Visit Schedule and Treatment Regimen

The following four diagnostic visits (Vs) were planned: V1 (screening; no anti-VEGF treatment), V2 (2–3 months after V1) after intravitreal anti-VEGF administration, V3 (6–8 months after V1 after intravitreal anti-VEGF administration), and V4 (12–14 months after V1 after intravitreal anti-VEGF administration), with measurements performed prior to any injection at that visit. Before each injection, we assessed visual acuity (decimal Snellen chart), intraocular pressure (IOP; non-contact air-puff tonometer, (Topcon, Healthcare, Tokyo, Japan CT-800), slit-lamp biomicroscopy (anterior segment, lens, and fundus), and OCT, (Copernicus plus, Optopol Technology S.A., Zawiercie, Poland) for central retinal thickness (CRT) and eye axial length (IOL Master 500, Carl Zeiss, Jena, Germany)). Of the 21 analysed patients, the drug was administered to the right eye in 16 (76.2%) and to the left eye in 5 (23.8%). During follow-up, 6 patients (28.6%) received treatment in the contralateral eye; by the end of the study, the untreated control eye remained the left eye in 10 (47.6%) and the right eye in 5 patients (23.8%). Between V1 and V2, 20 patients received one injection, and 1 patient received two injections; between V2 and V3, 10 received five injections, 9 received four, 1 received three, and 1 received two; between V3 and V4, 15 received two injections, 4 received three, and 2 had no treatment. For intravitreal injections we used aflibercept 2 mg, (Eylea, Bayer AG, Berlin, Germany) (in 16 patients, 8 female) or bevacizumab 1.25 mg (Avastin, F. Hoffman-La Roche AG, Basel, Switzerland) (in 5 patients, 3 female) (one drug type for one patient), with the fact that both drugs have the same therapeutic effect. The choice of drug was based on individual patient criteria and vision status.

### 2.4. Adaptive Optics (AO) Imaging Protocol

AO imaging (rtx1, Imagine Eyes, Orsay, France) was performed at V1–V4 without pharmacologic mydriasis in a dimly lit, air-conditioned room. After transitioning from standing, patients rested seated for 10–15 min to stabilise baroreflex-mediated hemodynamics prior to measurements. Photoreceptor analysis included the number of cones (N) and cone regularity (Reg) within a standardised 80 × 80 µm sampling window (manufacturer setting), recorded separately for each eye. Retinal arteriolar morphology was assessed in the superior temporal branch after the first bifurcation in the parapapillary region (right and left eyes) as follows: vessel diameter (VD), lumen diameter (LD), wall thickness (WT), wall-to-lumen ratio (WLR = WT/LD), and wall cross-sectional area (WCSA). For subsequent analyses, we used values from the arterial tree ipsilateral to the treated eye.

### 2.5. Peripheral and Central Hemodynamics

Brachial (peripheral) and central (aortic) pressures were obtained using SphygmoCor XCELL, AtCor Pty, Ltd., Sydney, Australia) at V1–V4 as follows: systolic (SBP, cSBP), diastolic (DBP, cDBP), mean arterial pressure (MAP, cMAP), and pulse pressure (PP, cPP). Waveform-derived indices included augmentation pressure (AP) and augmentation index (AIx = AP/PP, %). Aortic stiffness was assessed as carotid–femoral pulse wave velocity (PWV) and heart rate-adjusted PWV (PWVHR).

### 2.6. Carotid Ultrasound

Carotid ultrasound (performed with Samsung Medison Co., Ltd. SonoAce R7, Seoul, Republic of Korea) (right and left sides) was performed 2 mm proximal to the bifurcation during diastole to measure intima–media thickness (IMT), carotid lumen diameter (CLD), and the IMT/CLD ratio (IMTLR).

### 2.7. Outcomes

Predefined ocular endpoints included AO-derived WLR and LD in the treated eye, foveal cone number (N) and regularity (Reg), visual acuity (Visus), intraocular pressure (IOP), and central retinal thickness (CRT). Systemic endpoints comprised peripheral and central pressures, waveform indices (AP, AIx), aortic stiffness (PWV, PWVHR), and carotid IMT, CLD, and IMTLR.

### 2.8. Statistical Analysis

Analyses were conducted in IBM SPSS Statistics v29 (IBM Corp., Armonk, NY, USA). Continuous variables are summarised as mean ± standard deviation (SD) and median with interquartile range (IQR, 25th–75th percentile). For repeated measures across V1–V4, we applied Friedman tests. Pairwise comparisons of V2, V3, and V4 versus baseline (V1) were performed using Wilcoxon signed-rank tests. Two-tailed *p* < 0.05 was considered statistically significant. No formal multiplicity adjustment was applied to secondary endpoints.

## 3. Results

### 3.1. Baseline Clinical Characteristic of the Study Cohort

Table 1 shows basic demographic and laboratory data of study group.

At the first visit, patients who received bevacizumab according to the Mann–Whitney test were significantly older (68.9 ± 8.6 vs. 52.2 ± 16.2; *p* = 0.025), had higher HbA1c (8.99 ± 1.29 vs. 7.58 ± 1.34; *p* = 0.019), and a lower heart rate (70 ± 11 vs. 85 ± 10; *p* = 0.11), compared to patients who received aflibercept. Moreover, both groups did not differ significantly in the analysis of other parameters at the first visit, neither in the values of the examined clinical parameters, nor in the results of the retinal parameters in RTX1, nor in the results of the common carotid artery parameters in Doppler ultrasound.

### 3.2. Ocular (Local) Values

#### 3.2.1. Visual Acuity, Intraocular Pressure (Tonus), and Central Retinal Thickness (CRT)

**Figure 1:**
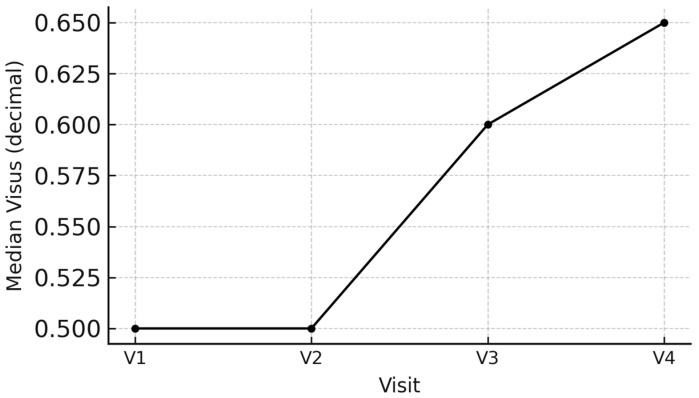
Changes in median values of the visual acuity in treated eyes (Visus TEs) over follow-up period.

**Figure 2:**
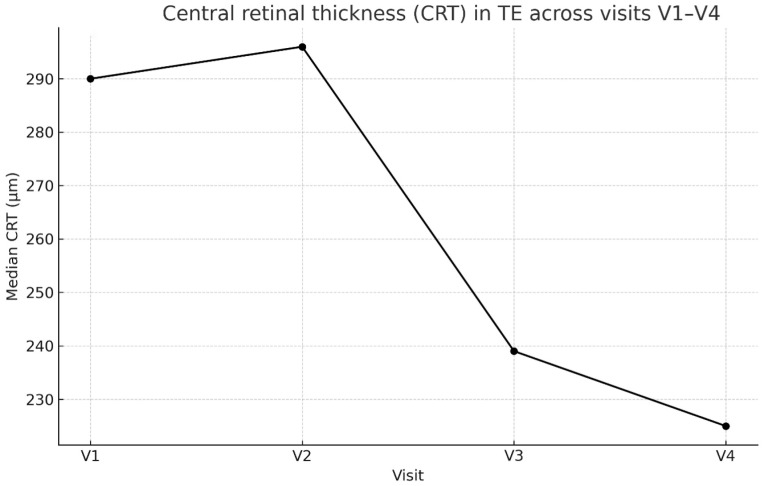
Changes in median retinal thickness (CRT) in the treated eye across visits V1–V4.

Visual acuity increased by 30% at V4 and CRT decreased by 22.4% at V4 significantly at V3 (Wilcoxon test: *p* = 0.009, *p* = 0.002, respectively) and V4 (Wilcoxon test: *p* = 0.028, *p* = 0.030, respectively) compared with baseline (Table 2 and Table 3). Tonus changes were not significant (Table 4) (Figure 1 and Figure 2). Tables S1 and S2 with data associated with Table 2 and Table 3.

##### Adaptive Optics (AO) Imagining Protocol: Photoreceptors Analysis

Cone regularity (Reg) improved significantly at V4 post-therapy compared with pre- therapy V1 (*p* = 0.018) by 27.5%. The number of visible cones increased more than double in Visit 3 and 4 compared to V1, but this increase is significant (*p* = 0.012) in comparison of V3 to V1 (Table 5, Table 6 and Table 7). Tables S3 and S4 with data associated with Table 5.

##### Adaptative Optics (AO) Imagining Protocol: Retinal Arteriolar Morphology

Analysis of morphological and functional changes in retinal blood vessels are shown in Table 8. Tables S5 and S6 with data associated with Table 8.

Significant (*p* < 0.05) changes in retinal arteriole lumen diameter (LD) and wall-to-lumen ratio (WLR) were observed in treated eyes (TEs) (Table 9).

**Figure 3:**
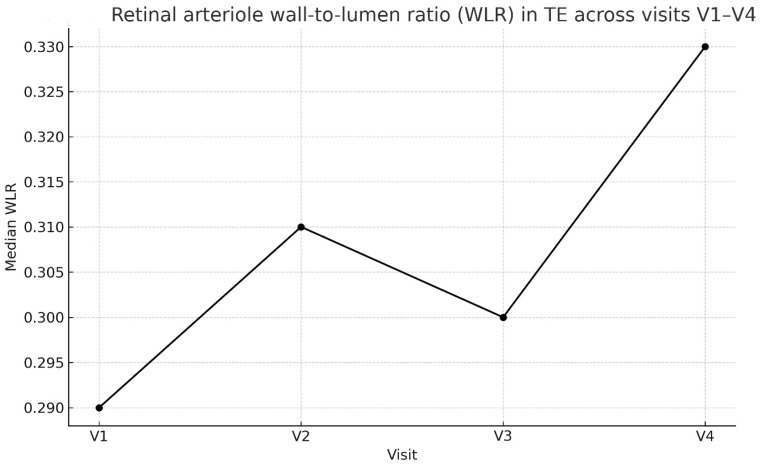
Retinal arteriole wall-to lumen ratio (WLR) in treated eye across visits V1–V4.

In treated and in control eyes, WLR and LD values were compared by Wilcoxon test between the V2–V4 post-drug visits and the V1 pre-drug examination. The median of wall-to-lumen ratio in the treated eye was significantly (*p* = 0.045) higher by 13.8% in Visit 4, compared to Visit 1 measured in treated eyes but not in control eyes (Figure 3). The lumen diameter did not change significantly in both eyes during the post-drug visits compared to pre-drug examination.

The remaining morphological parameters of the retinal arterioles did not show any significant changes during the study.

### 3.3. Systemic Values

#### 3.3.1. Analysis of Morphological and Functional Changes in Major Blood Vessels

##### Morphological Analysis of Major Blood Vessels

In both carotid arteries, neither on the side of the treated eye nor on the control side no significant longitudinal morphological changes in carotid artery were observed, confirming systemic vascular safety (Table 10). Tables S7–S9 with data associated with Table 10.

##### Functional Analysis of Major Blood Vessels

**Figure 4:**
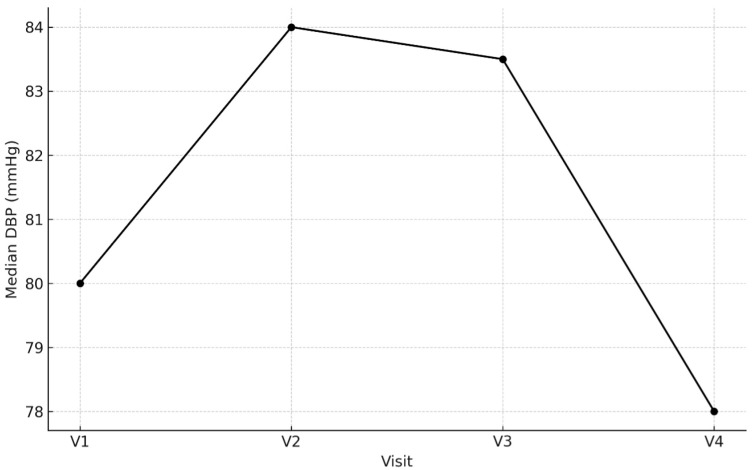
Peripheral diastolic blood pressure across V1–V4.

We observed significant differences in the DBP (*p* = 0.034) and cDBP (*p* = 0.022) tests using the Friedman test when compared to V1, but they were not confirmed as significant when comparing subsequent measurements after drug administration to those before drug administration in Wilcoxon test (*p* > 0.05) (Figure 4), with the exception of the significant reduction in DBP and cDBP that was observed at V4 compared with baseline (*p* < 0.05) only. Detailed characteristic of central and peripheral hemodynamic parameters of the major vessels, heart rhythm and aortic stiffness are presented in Table 11. Tables S10–S12 with data associated with Table 11.

## 4. Discussion

In this prospective observational study, we comprehensively evaluated retinal and systemic vascular changes in patients with diabetic macular oedema (DME) undergoing anti-VEGF therapy. Our findings demonstrated that intravitreal administration of aflibercept or bevacizumab was associated with measurable changes in microvascular retinal parameters, including wall-to-lumen ratio (WLR) and improvements of the cone regularity, as well as reductions in central retinal thickness (CRT) and consequently in visual acuity improvement. Importantly, systemic vascular indices, such as carotid artery morphology and central aortic stiffness, remained stable, suggesting that anti-VEGF therapy is safe for large vessels. These results are consistent with previous reports showing that anti-VEGF treatment effectively reduces retinal oedema and improves visual function in DME patients [1,2,3]. Our analysis expands upon earlier findings by providing adaptive optics and Doppler ultrasound data, suggesting that structural changes occur at the retinal microvascular level while the integrity of large systemic vessels appears to be preserved.

Notably, a significant improvement in cone regularity in diabetes mellitus [17] and partial recovery of cone number were observed during follow-up, highlighting the potential of anti-VEGF therapy not only to preserve but also to restore retinal photoreceptor morphology.

Previous studies using adaptive optics have emphasised the relevance of microvascular parameters, such as WLR, in assessing early vascular remodelling in systemic diseases [4,5,6,18]. Our findings confirm that WLR increased after treatment, which may reflect changes in retinal vascular structure (WLR) under anti-VEGF therapy.

In contrast, no significant changes were noted in common carotid artery intima–media thickness or lumen diameter, further reinforcing the systemic safety of this therapy.

**Figure 5:**
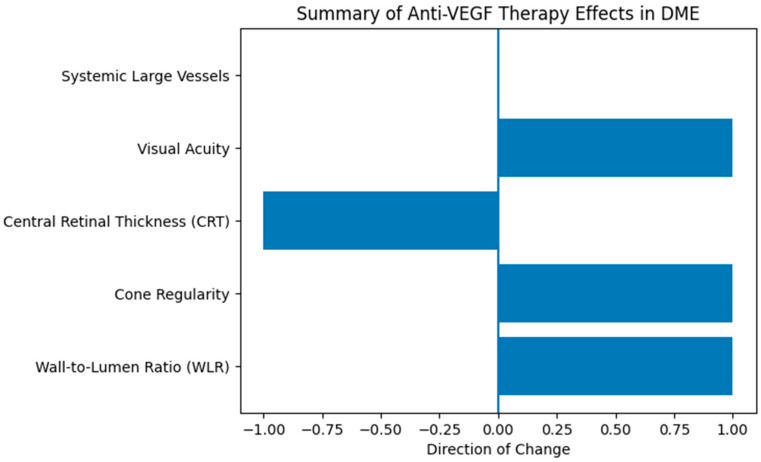
Graphical summary of retinal and systemic vascular changes after anti-VEGF treatment.

Directional changes in retinal microvascular, structural, and systemic vascular parameters following anti-VEGF therapy are shown in Figure 5.

This study primarily focused on morphological changes in retinal vessels as well as the morphology and function of large peripheral vessels and did not assess vascular events related to intravitreal anti-VEGF administration. On the other hand, previous reports have suggested a potential association between intravitreal anti-VEGF therapy and an increased risk of ischemic cardiac events [19,20]. Importantly, existing evidence also indicates that intravitreal administration of anti-VEGF agents is associated with a vascular event risk comparable to that observed in patients not receiving anti-VEGF therapy, suggesting no significant increase in systemic vascular risk attributable to these treatments [21,22,23]. Evaluation of such clinical outcomes was beyond the scope of the present study.

Nevertheless, our study has certain limitations. The relatively small sample size and single-centre design limit the generalizability of the results. Moreover, the observational nature of the study precludes definitive causal inferences. Larger multicentre trials with longer follow-up periods are warranted to validate these findings and to further investigate the long-term systemic vascular safety of anti-VEGF treatment in diabetic populations.

Another limitation of this study is the use of two different anti-VEGF agents, bevacizumab and aflibercept; however, the primary objective was to evaluate the overall effects of anti-VEGF therapy on retinal and systemic vasculature and retinal structure rather than the effects of a specific agent. Patients received these medications a varying number of times between visits, as required by therapeutic necessity, which may have influenced the outcomes. A direct comparison of the effects of these drugs would require a larger, dedicated patient cohort.

Patients who develop diabetic complications in the retina, most often due to complications such as cataracts, have eyes with low transparency of optical media, which is necessary for non-invasive testing using modern technology such as RTX1. Collecting enough patients for statistical testing took two years and was interrupted due to the SARS-CoV-2 pandemic. Because the two groups did not differ significantly in the analysis of most clinical parameters at the first visit, particularly in the results of retinal parameters using RTX1 and the results of Doppler ultrasound of the common carotid artery, both groups were included into a single group treated with anti-VEGF medications, and the statistical analysis was based on the group.

## 5. Conclusions

Intravitreal anti-VEGF therapy in patients with diabetic macular oedema led to significant improvements in retinal microvascular structure, including reductions in central retinal thickness and improvements in cone regularity and number of visible cones. We observed a significant increase in wall-to-lumen ratio in retinal arterioles under the influence of anti-VTGF therapy, which requires further investigation. Importantly, systemic vascular parameters, including carotid artery morphology and central aortic stiffness, remained unaffected, indicating the systemic safety of the treatment.

Our findings support the role of anti-VEGF therapy as an effective and safe intervention for DME. However, the study’s limitations, particularly the small sample size, highlight the need for further large-scale studies to confirm these results and evaluate long-term outcomes.

## Figures and Tables

**Table 1:** Baseline clinical characteristics of the study cohort.

Clinical Parameter	Mean	SD	Median	IQR 25th	IQR 75th
Age (years)	64.95	12.71	68.0	59.0	72.5
Axial length TE (mm)	23.33	0.64	23.25	22.88	22.82
Axial length KE (mm)	23.26	0.61	23.21	23.64	23.65
Weight (kg)	84.37	15.0	81.4	74.75	94.8
Height (m)	1.67	0.10	1.68	1.59	1.73
BMI (kg/m^2^)	30.29	4.83	29.92	27.53	32.39
Waist (cm)	108.67	12.53	108.0	100.5	116.0
Creatinine (mg/dL)	1.30	0.86	1.10	0.75	1.40
eGFR (mL/min/1.73 m^2^)	62.21	28.10	59.05	45.03	81.33
Cholesterol (mg/dL)	167.24	54.67	165.0	128.0	188.0
Triglycerides (mg/dL)	147.05	139.71	112.0	83.0	158.5
HDL (mg/dL)	56.52	14.80	57.0	46.0	68.5
LDL (mg/dL)	97.00	41.74	93.0	67.5	130.0
HbA1c (%)	8.65	1.41	8.70	8.20	9.55
Random glycemia (mg/dL)	178.11	50.39	185.5	140.0	215.0

**Table 2:** Visual acuity (Visus), tonus, and central retinal thickness (CRT) in the treated eye during Visit 1.

Parameter	Unit	Mean	SD	Median	IQR
Visus_TE	Ratio	0.52	0.23	0.50	0.30–0.70
Tonus_TE	[mmHg]	16.1	2.4	17.0	15.0–18.0
CRT_TE	[µm]	318	102	290	253–375

**Table 3:** Visual acuity (Visus), intraocular pressure (tonus), and central retinal thickness (CRT) in the treated eye during Visit 4.

Parameter	Unit	Mean	SD	Median	IQR
Visus_TE	Ratio	0.65	0.25	0.60	0.48–0.85
Tonus_TE	[mmHg]	17.2	1.3	17.0	17.0–18.3
CRT_TE	[µm]	266	108	225	205–280

**Table 4:** Wilcoxon test for visual acuity, central retinal thickness, and intraocular pressure (tonus) changes over the study period.

Parameter	*p* V1/V2	*p* V1/V3	*p* V1/V4
Visus_TE	0.29	**0.009**	**0.028**
Tonus_TE	0.73	0.62	0.44
CRT_TE	0.99	**0.002**	**0.030**

**Table 5:** Cone number and regularity in both treated eye (TE) and control eye (KE) in Visit 1.

Parameter	Unit	Mean	SD	Median	IQR
N_TE	number	32.2	28.1	26.0	6.5–50.5
N_KE	number	45.2	27.8	51.0	14.5–74.5
Reg_TE	%	80.3	17.2	86.3	65.7–92.3
Reg_KE	%	81.3	14.3	84.9	72.3–93.3

**Table 6:** Cone number and regularity in both treated eye (TE) and control eye (KE) in Visit 4 (with Friedman test), with comparison of the parameters by Friedman test measured during the experiment duration in Visit 1–4.

Parameter	Unit	Mean	SD	Median	IQR	*p* (Friedman Test V1–V4)
N_TE	number	56.9	35.7	60.0	20.5–90.5	0.095
N_KE	number	45.7	41.7	39.5	8.3–90.0	0.69
Reg_TE	%	91.1	4.2	90.8	89.5–93.6	0.019
Reg_KE	%	84.5	17.0	91.9	71.4–94.0	0.28

**Table 7:** Wilcoxon test for number and cone regularity.

Parameter	*p* V1/V2	*p* V1/V3	*p* V1/V4
N_TE	0.78	**0.012**	0.17
Reg_TE	0.74	0.29	**0.018**
N_KE	0.26	0.53	0.35
Reg_KE	0.24	0.65	0.89

**Table 8:** Retinal arteriole morphology parameters in both treated eye (TE) and control eye (KE) in Visit 1.

Parameter	Unit	Mean	SD	Median	IQR
VD_TE	µm	126.39	17.74	129.8	113.9–139.3
LD_TE	µm	98.12	14.71	99.0	85.6–109.7
WT_TE	µm	14.49	2.70	14.7	13.0–15.5
WLR_TE	Ratio	0.30	0.05	0.29	0.27–0.34
WCSA_TE	µm^2^	5169	1471	5229	4167–6172
VD_KE	µm	126.12	11.97	129.0	116.7–137.7
LD_KE	µm	92.62	9.05	94.1	85.5–99.7
WT_KE	µm	16.75	2.89	17.9	14.4–19.1
WLR_KE	Ratio	0.36	0.07	0.36	0.30–0.42
WCSA_KE	µm^2^	5797	1315	5907	4807–6878

**Table 9:** Wilcoxon test for retinal arterioles morphology parameters.

Parameter	*p* V1/V2	*p* V1/V3	*p* V1/V4
WLR_TE	0.052	0.48	0.045
LD_TE	0.24	0.50	0.29
WLR_KE	0.14	0.61	0.50
LD_KE	0.77	0.89	0.83

**Table 10:** Morphological parameters of the common carotid artery during the experimental Visit 1.

Parameter	Mean	SD	Median	IQR
IMT_TS [cm]	0.09	0.03	0.10	0.08–0.11
CLD_TS [cm]	0.66	0.09	0.65	0.59–0.75
IMTLR_TS (Ratio)	0.14	0.04	0.14	0.11–0.18
IMT_KS [cm]	0.09	0.03	0.09	0.07–0.11
CLD_KS [cm]	0.64	0.11	0.66	0.57–0.74
IMTLR_KS (Ratio)	0.14	0.04	0.14	0.11–0.17

**Table 11:** Hemodynamic parameters—peripheral and central blood pressure, heart rate, and aortic stiffness during Visit 1.

Parameter	Mean	SD	Median	IQR
SBP [mmHg]	149.86	23.04	151.0	133.5–164.0
DBP [mmHg]	80.90	12.02	80.0	70.0–91.5
MAP [mmHg]	103.89	13.97	105.0	93.0–116.5
PP [mmHg]	68.95	18.76	64.0	54.0–80.5
HR [bpm]	73.38	12.18	74.0	65.0–82.0
cSBP [mmHg]	133.90	18.88	133.0	120.5–146.0
cDBP [mmHg]	81.81	12.21	81.0	70.5–93.0
cMAP [mmHg]	102.57	13.90	102.0	91.5–116.0
cPP [mmHg]	49.05	17.98	48.0	38.0–63.0
AP [mmHg]	13.52	8.94	17.0	7.0–20.5
AIx [%]	24.14	14.41	26.0	16.5–35.0
PWV [m/s]	10.62	2.71	10.9	8.9–12.3
PWVHR [bpm]	69.10	11.70	71.0	61.0–77.0

## Data Availability

No new data were created or analyzed in this study. Data sharing is nor applicable to this article.

## Supplementary Materials

The following supporting information can be downloaded at https://www.mdpi.com/article/10.3390/biomedicines14010124/s1; Table S1: Visual Acuity (Visus), Tonus and Central retinal Thickness (CRT) in Treated Eye during the Visit 2; Table S2: Visual Acuity (Visus), Tonus and Central retinal Thickness (CRT) in Treated Eye during the Visit 3; Table S3: Cone Number and Regularity in both treated eye (TE) and control eye (KE) in Visit 2; Table S4: Cone Number and Regularity in both treated eye (TE) and control eye (KE) in Visit 3; Table S5: Retinal arteriole morphology parameters in both treated eye (TE) and control eye (KE) in Visit 3; Table S6: Retinal arteriole morphology parameters in both treated eye (TE) and control eye (KE) in Visit 4 with comparison of the parameters by Friedman Test measured during the experiment duration in Visit1–4; Table S7: Morphological parameters of the common carotid artery during the experiment Visit 2; Table S8: Morphological parameters of the common carotid artery during the experiment Visit 3; Table S9: Morphological parameters of the common carotid artery during the experiment in Visit 4 with comparison of the parameters by Friedman Test measured during the experiment duration in Visit1–4; Table S10: Hemodynamic Parameters—Peripheral and Central Blood Pressure, Heart Rate, and Aortic Stiffness During Visit 2; Table S11: Hemodynamic Parameters—Peripheral and Central Blood Pressure, Heart Rate, and Aortic Stiffness During Visit 3; Table S12: Hemodynamic Parameters—Peripheral and Central Blood Pressure, Heart Rate, and Aortic Stiffness During Visit 4.

## Abbreviations

The following abbreviations are used in this manuscript:

AIx	Augmentation index
AP	Aortic augmented pressure
AxL_KE	Axial length, control eye
AxL_TE	Axial length, treated eye
BMI	Body mass index
CLD	Carotid lumen diameter
CRT	Central retinal thickness
DBP	Diastolic blood pressure (brachial)
cDBP	Central diastolic blood pressure (aortic)
eGFR	Estimated glomerular filtration rate
HbA1c	Glycated haemoglobin
HDL	High-density lipoprotein cholesterol
HR	Heart rate
IMT	Intima–media thickness
IMTLR	Intima/media thickness-to-lumen ratio
IOP (Tonus)	Intraocular pressure
IQR	Interquartile range
LD	Lumen diameter (retinal arteriole)
LDL	Low-density lipoprotein cholesterol
MAP	Mean arterial pressure (brachial)
cMAP	Central mean arterial pressure (aortic)
N	Number of cones within a standardised 80 × 80 µm sampling window
PP	Pulse pressure (PP = Sys − Dia)
cPP	Central pulse pressure (cPP = cSys − cDia)
PWV	Carotid–femoral pulse wave velocity
PWVHR	Heart rate-adjusted PWV
Reg	Cone regularity within a standardised 80 × 80 µm window
SBP	Systolic blood pressure (brachial)
cSBP	Central systolic blood pressure (aortic)
TE	Treated eye
KE	Control eye
TGL	Triglycerides
VD	Vessel diameter (retinal arteriole)
Visus	Visual acuity
WLR	Wall-to-lumen ratio
WT	Wall thickness (retinal arteriole)
WCSA	Wall cross-sectional area (retinal arteriole)
