# Supplementary material for: Retinal Organisation and Systemic Vascular Changes Assessed by Adaptive Optics and Doppler Ultrasonography Following Anti-VEGF Therapy in Patients with Diabetic Macular Oedema
Source: Biomedicines. 2026 Jan 8;14(1):124. doi: 10.3390/biomedicines14010124 (PMC12839161; doi:10.3390/biomedicines14010124)
Supplement: Supplementary file 1 [file biomedicines-14-00124-s001.zip › biomedicines-4019323-supplementary.pdf]

Supplementary tables (not included in the main text).

1. Supplementary tables with data associated with Tables 2 and 3.

Table S1. Visual Acuity (Visus), Tonus and Central retinal Thickness (CRT) in Treated Eye during the Visit 2.

Parameter	Unit	Mean	SD	Median	IQR
Visus_TE	Ratio	0.50	0.23	0.50	0.35 – 0.65
Tonus_TE	[mmHg]	16.3	2.8	16.0	14.0 – 18.0
CRT_TE	[µm]	314	114	296	225 – 376

Table S2. Visual Acuity (Visus), Tonus and Central retinal Thickness (CRT) in Treated Eye during the Visit 3.

Parameter	Unit	Mean	SD	Median	IQR
Visus_TE	Ratio	0.62	0.25	0.60	0.40 – 0.80
Tonus_TE	[mmHg]	16.4	1.4	16.5	15.3 – 17.8
CRT_TE	[µm]	255	63	239	207 – 302

2. Supplementary tables with data associated with Tables 5.

Table S3. **Cone Number and Regularity in both treated eye (TE) and control eye (KE) in Visit 2.**

Parameter	Unit	Mean	SD	Median	IQR
	number	34.6	25.3	27.5	15.0 – 56.3
N_KE	number	48.7	30.1	55.0	20.0 – 71.0
Reg_TE	%	76.1	23.0	85.3	65.5 – 89.8
Reg_KE	%	86.3	6.6	86.8	82.0 – 92.1

Table S4**. Cone Number and Regularity in both treated eye (TE) and control eye (KE) in Visit 3**

Parameter	Unit	Mean	SD	Median	IQR
N_TE	number	55.4	25.7	56.5	39.3 – 73.3
N_KE	number	48.8	29.2	52.0	22.0 – 68.0
Reg_TE	%	86.9	8.6	87.5	81.5 – 94.7
Reg_KE	%	81.3	23.0	88.0	80.5– 94.7

3. Supplementary tables with data associated with Tables 5.

Table S5. Retinal arteriole morphology parameters in both treated eye (TE) and control eye (KE) in Visit 3

Parameter	Unit	Mean	SD	Median	IQR
VD_TE	µm	125.23	17.25	127.4	114.4 – 139.8
LD_TE	µm	96.84	15.10	94.9	86.8 – 111.9
WT_TE	µm	14.19	2.40	12.3	14.2 – 17.0
WLR_TE	Ratio	0.30	0.05	0.30	0.25 – 0.34
WCSA_TE	µm²	5003	1299	4850	4301 – 5897
VD_KE	µm	122.68	10.74	121.5	113.8 – 131.7
LD_KE	µm	91.13	9.66	91.1	84.5 – 97.7
WT_KE	µm	15.78	2.13	16.1	13.8 – 17.9
WLR_KE	Ratio	0.35	0.06	0.34	0.31 – 0.39
WCSA_KE	µm²	5313	958	5443	4436 – 5969

Table S6. Retinal arteriole morphology parameters in both treated eye (TE) and control eye (KE) in Visit 4 with comparison of the parameters by Friedman Test measured during the experiment duration in Visit1-4.

Parameter	Unit	Mean	SD	Median	IQR	p (Friedman Test V1–V4)
VD_TE	µm	129.58	16.36	130.6	113.1 – 141.6	0.096
LD_TE	µm	98.23	14.27	99.2	83.1 – 109.6	0.034
WT_TE	µm	15.67	2.11	15.7	14.7 – 16.4	0.29
WLR_TE	Ratio	0.32	0.05	0.33	0.27 – 0.36	0.007
WCSA_TE	µm²	5653	1306	5476	4927 – 6578	0.79

VD_KE	µm	122.18	11.05	119.5	113.8 – 134.4	0.10
LD_KE	µm	89.13	10.66	85.5	81.2 – 99.8	0.46
WT_KE	µm	16.04	1.92	15.9	14.1 – 18.0	0.52
WLR_KE	Ratio	0.36	0.06	0.37	0.32 – 0.42	0.54
WCSA_KE	µm²	5347	840	5274	4520 – 6000	0.98

4. Supplementary tables with data associated with Tables 10.

Table S7. Morphological parameters of the common carotid artery during the experiment Visit 2.

Parameter	Mean	SD	Median	IQR
IMT_TS [cm]	0.08	0.02	0.08	0.07–0.10
CLD_TS [cm]	0.67	0.09	0.68	0.62–0.74
IMTLR_TS (Ratio)	0.13	0.04	0.11	0.10–0.16
IMT_KS [cm]	0.08	0.02	0.08	0.07–0.10
CLD_KS [cm]	0.67	0.09	0.68	0.57–0.75
IMTLR_KS (Ratio)	0.13	0.04	0.12	0.11–0.15

Table S8. Morphological parameters of the common carotid artery during the experiment Visit 3.

Parameter	Mean	SD	Median	IQR
IMT_TS [cm]	0.09	0.03	0.09	0.08–0.12
CLD_TS [cm]	0.66	0.08	0.65	0.60–0.73
IMTLR_TS (Ratio)	0.14	0.04	0.13	0.11–0.16
IMT_KS [cm]	0.09	0.03	0.08	0.07–0.12
CLD_KS [cm]	0.66	0.08	0.68	0.59–0.70
IMTLR_KS (Ratio)	0.14	0.04	0.14	0.10–0.17

Table S9. Morphological parameters of the common carotid artery during the experiment in Visit 4 with comparison of the parameters by Friedman Test measured during the experiment duration in Visit1-4.

Parameter	Mean	SD	Median	IQR	p (Friedman Test) Visit1–4
IMT_TS [cm]	0.08	0.03	0.08	0.07–0.10	0.48
CLD_TS [cm]	0.65	0.11	0.68	0.54–0.74	0.39
IMTLR_TS (Ratio)	0.13	0.04	0.12	0.11–0.16	0.71
IMT_KS [cm]	0.09	0.03	0.09	0.07–0.11	0.80
CLD_KS [cm]	0.61	0.08	0.61	0.53–0.66	0.25
IMTLR_KS (Ratio)	0.15	0.05	0.14	0.11–0.17	0.70

5. Supplementary tables with data associated with Tables 11.

Table S10**.** Hemodynamic Parameters — Peripheral and Central Blood Pressure, Heart Rate, and Aortic Stiffness During Visit 2.

Parameter	Mean	SD	Median	IQR
SBP [mmHg]	156.24	18.61	152.0	141.5–172.5
DBP [mmHg]	83.24	9.96	84.0	73.0–92.0
MAP [mmHg]	107.57	11.36	107.3	97.3–119.7
PP [mmHg]	73.00	15.39	69.0	59.5–83.5
HR [bpm]	72.76	10.32	74.0	63.5–79.5
cSBP [mmHg]	138.48	15.01	136.0	127.5–149.5
cDBP [mmHg]	84.57	9.96	88.0	75.0–93.5
cMAP [mmHg]	105.95	10.93	105.0	97.0–117.5
cPP [mmHg]	55.33	13.77	53.0	42.5–65.0
AP [mmHg]	13.86	9.06	14.0	5.5–19.5
AIx [%]	23.05	13.61	24.0	12.5–34.0
PWV [m/s]	10.98	2.12	10.9	9.1–12.5
PWVHR [bpm]	70.95	9.37	71.0	65.0–78.5

Table S11. Hemodynamic Parameters — Peripheral and Central Blood Pressure, Heart Rate, and Aortic Stiffness During Visit 3.

Parameter	Mean	SD	Median	IQR
SBP [mmHg]	155.50	21.75	151.0	141.0–168.8
DBP [mmHg]	84.60	12.75	83.5	75.3–94.3
MAP [mmHg]	108.23	13.41	110.5	97.8–116.3

PP [mmHg]	70.90	19.70	67.0	58.3–76.5
HR [bpm]	74.85	9.79	77.0	66.3–80.0
cSBP [mmHg]	138.85	18.11	136.5	125.0–152.5
cDBP [mmHg]	85.80	12.67	84.5	75.3–95.0
cMAP [mmHg]	107.15	13.45	110.5	96.3–116.5
cPP [mmHg]	51.65	18.59	53.0	39.5–64.0
AP [mmHg]	13.05	9.82	14.0	6.0–20.5
AIx [%]	23.55	15.92	27.0	14.8–33.5
PWV [m/s]	11.17	1.89	11.2	10.3–12.1
PWVHR [bpm]	71.55	9.53	70.0	65.0–80.5

Table S12. Hemodynamic Parameters — Peripheral and Central Blood Pressure, Heart Rate, and Aortic Stiffness During Visit 4.

Parameter	Mean	SD	Median	IQR
SBP [mmHg]	146.00	14.97	145.0	134.0–158.0
DBP [mmHg]	79.93	10.69	78.0	72.0–86.0
MAP [mmHg]	101.96	10.46	100.0	94.7–108.0
PP [mmHg]	66.07	13.69	64.0	56.0–74.0
HR [bpm]	74.47	6.78	76.0	69.0–80.0
cSBP [mmHg]	129.80	12.44	129.0	119.0–139.0
cDBP [mmHg]	80.67	10.59	78.0	73.0–86.0
cMAP [mmHg]	100.67	10.77	97.0	93.0–108.0
cPP [mmHg]	49.07	11.60	44.0	41.0–60.0
AP [mmHg]	11.33	7.74	12.0	4.3–22.0
AIx [%]	22.73	12.82	25.0	15.0–30.0
PWV [m/s]	10.14	1.48	10.0	9.0–10.9
PWVHR [bpm]	71.29	6.93	71.0	65.8–76.3
